# Antibodies in the breastmilk of COVID-19 recovered women

**DOI:** 10.1186/s12884-022-04945-z

**Published:** 2022-08-11

**Authors:** Paulina Szczygioł, Błażej Łukianowski, Katarzyna Kościelska-Kasprzak, Katarzyna Jakuszko, Dorota Bartoszek, Magdalena Krajewska, Barbara Królak-Olejnik

**Affiliations:** 1 Department of Neonatology, Wroclaw Medical University, 50-367 Wroclaw, Poland; 2 Department of Nephrology and Transplantation Medicine, Wroclaw Medical University, 50-367 Wroclaw, Poland

**Keywords:** Human milk, Breastfeeding, SARS-CoV-2, Immunoglobulins, passive immunity

## Abstract

**Objective:**

Human milk contains antibodies against Severe Acute Respiratory Syndrome Coronavirus 2 (SARS-CoV-2) which may serve as a protective factor through passive immunization in infants. The objective of this study was to measure the levels of anti-SARS-CoV-2 IgG and IgA in human milk and serum after a SARS-CoV-2 infection.

**Design:**

Breast milk and serum samples from 72 lactating mothers with confirmed SARS-CoV-2 asymptomatic or symptomatic infection were collected 1-229 days after the onset of clinical symptoms related to COVID-19. Seventeen mothers with no history of COVID-19 served as a control group. Enzyme-Linked ImmunoSorbent Assay was performed to analyze antibodies against SARS-CoV-2.

**Results:**

SARS-CoV-2-IgA human milk antibodies were detected in mothers and their concentrations were consistently higher than SARS-CoV-2-IgG antibodies. The serum and breastmilk samples of women with COVID-19 was characterized by a higher concentration of anti-RBD IgA and IgG than the serum from the control group without COVID-19. No statistically significant difference was observed between the antibody levels in the serum samples obtained from symptomatic and asymptomatic women exposed to SARS-CoV-2 and between the antibody level and the time from a positive SARS-CoV-2 test result over the period studied.

**Conclusion:**

Our results confirm the presence of SARS-CoV-2 IgA and IgG antibodies in the breastmilk of COVID-19 recovered women and the possibility of these antibodies in providing specific immunologic benefits to breastfeeding infants such as protection against the virus transmission and severity of the acquired COVID-19 disease.

**Supplementary Information:**

The online version contains supplementary material available at 10.1186/s12884-022-04945-z.

## Introduction

The ongoing global pandemic of coronavirus disease 2019 (COVID-19) raised the questions about newborn infants’ protection against respiratory infection caused by severe acute respiratory coronavirus 2 (SARS-CoV-2). Due to the immaturity of the immune system, infants represent a unique at-risk population for which SARS-CoV-2 vaccines are not intended [1]. The main source of newborns’ passive immunity is maternal milk [2]. The benefits of breastfeeding are well established, therefore even in case of COVID-19 disease, the World Health Organization (WHO, 2020) strongly recommends that women should be encouraged and supported to breastfeed [3]. Transmission of SARS-CoV-2 via breast milk is rare. A meta-analysis in 2020 showed that SARS-CoV-2 genome is generally not found in human milk of COVID-19 infected lactating women [4]. Human milk, as previous studies reported, is a source of maternal SARS-CoV-2 antibodies, which may serve as protection against SARS-CoV-2 [5–7]. However, research on immunoglobulins in human milk derived from women affected by COVID-19 during pregnancy are mostly limited due to small group size or insufficient information about milk collection and its analysis. The purpose of this study was to investigate the concentrations of SARS-CoV-2 IgG and IgA in human milk and serum and factors that may affect their levels. We performed Enzyme-Linked ImmunoSorbent Assay of blood serum and human milk from maternity patients who had recovered from COVID-19 at different trimesters of pregnancy and from mothers who had COVID-19 during the time of delivery.

## Materials and methods

### Study population

The study involved 72 women of which 54 had a history of COVID-19 at various trimesters of their pregnancy (11 1st trimester, 8 2nd trimester, 35 3rd trimester) and 18 women with an active COVID-19 during delivery. Women were patients at the Wroclaw Medical Teaching Hospital or were recruited via social media during the 3rd wave of coronavirus pandemic between 15 February to 1 May 2021. The inclusion criteria were as follows: PCR-confirmed coronavirus infection during pregnancy or delivery and lactation in the postpartum period. Seventeen women with no history of COVID-19 and any recent symptoms consistent with the infection constituted a control group. All women were seronegative for anti-SARS-CoV-2 antibodies. None of the study participants nor controls has been anti-SARS-CoV-2 vaccinated at the time of the study. All women gave written informed consent before study procedures were performed. Data regarding mothers and their infants were collected at the time of enrollment by filling the personal questionnaire.

### Serum and human milk collection

Maternal blood (5 ml) and breast milk (10 – 15 ml) samples were collected on the same day. Whole blood samples were left to clot at room temperature (15 min.) and then centrifuged at 1000 x g for 15 minutes. Subsequent serum was aliquoted and frozen at − 80 °C. Human milk samples were collected:

(I) at home and transported in insulated boxes up to 4 h to hospital (recruitment via social media)
(II) or in the hospital (recruitment in hospital).

Serum and breast milk samples were collected 1-229 (median 68) days after the patients tested positive for COVID-19. All mothers were instructed to express milk with clean electric breast pumps into sterile plastic containers after feeding the baby. Then, received milk samples were separated into aliquots, frozen and stored at − 80 °C until the ELISA measurements.

### Measurement of SARS-CoV-2-specific antibodies

The anti-SARS-CoV-2 immune response was assessed by determining the presence of anti-SARS-CoV-2 IgG and IgA antibodies in the maternal serum and breastmilk samples. The detection of antibodies against SARS-CoV-2 was based on the S1 domain of the spike protein including the immunologically relevant receptor binding domain (RBD) as an antigen. RBD represents important target antigen for virus neutralizing antibodies. The concentration of serum anti-SARS-CoV-2 IgG was measured using semi-quantitative enzyme linked immunosorbent assay (anti-SARS-CoV-2 IgG ELISA, #EI 2606-9601 G, Euroimmun, Germany). The concentration of breastmilk anti-SARS-CoV-2 IgG were measured using semi-quantitative enzyme linked immunosorbent assay (Euroimmun) and quantitative enzyme linked immunosorbent assay (anti-SARS-CoV-2 QuantiVac ELISA, #EI 2606-9601-10 G, Euroimmun, Germany). Serum samples were diluted 101 times as suggested by the manufacturer, while breastmilk samples were diluted 5 times. The breastmilk samples were vortexed for 3 minutes immediately before sample dilution. The presence of serum and breastmilk anti-SARS-CoV-2 IgA was measured using semi-quantitative ELISA (anti-SARS-CoV-2 IgA ELISA, #EI 2606-9601 A, Euroimmun, Germany). Serum samples were diluted 101 times as suggested by the manufacturer, while breastmilk samples were diluted 5 times. The breastmilk samples were vortexed for 3 minutes immediately before sample dilution. The quantitative test results of the measurement were converted to standardized binding antibody units (BAU/ml), which are in agreement with the First WHO International Standard Anti-SARS-CoV-2 Immunoglobulin (NIBSC 20/136). According to manufacturer guidelines, the results of semi-quantitative tests are presented as a ratio to the cut-off value, with the following interpretation of the results for the serum samples: < 0.8 interpreted as negative, ≥0.8 and < 1.1 borderline, and ≥ 1.1 positive. No cut-off values for breastmilk samples are available in the manufacturer guidelines. The measurements were performed on the coded samples, by researchers blinded to the information of the participant status. The participants were called seropositive, if they presented anti-SARS-CoV-2 IgG or IgA level defined positive according to manufacturer guidelines. If serum levels of both anti-SARS-CoV-2 IgG and IgA antibodies were below detection limit a participant was called seronegative.

### Statistical analysis

The descriptive statistics of variables is presented as mean with standard deviation (SD) and median with interquartile range (IQR). The normality of the distribution was checked using the Shapiro-Wilk test. All presented comparisons and correlations included not normally distributed variables. Intergroup comparisons of continuous data was assessed using the non-parametric Mann-Whitney U test or Wilcoxon signed-rank test for dependent variables. The correlations were performed with using rang correlation (Spearman). Receiver operating characteristic (ROC) curve was used to define the cut-off value for breastmilk IgG and IgA level related to former COVID-19. The results with *p* <  0.05 were considered significant. Statistical analysis was performed using the STATISTICA 13 statistical package (TIBCO Software Inc. Palo Alto, CA, USA) and MedCalc (version 20, MedCalc Software).

## Results

### Basic information of mothers and their neonates

**Fig. 1:**
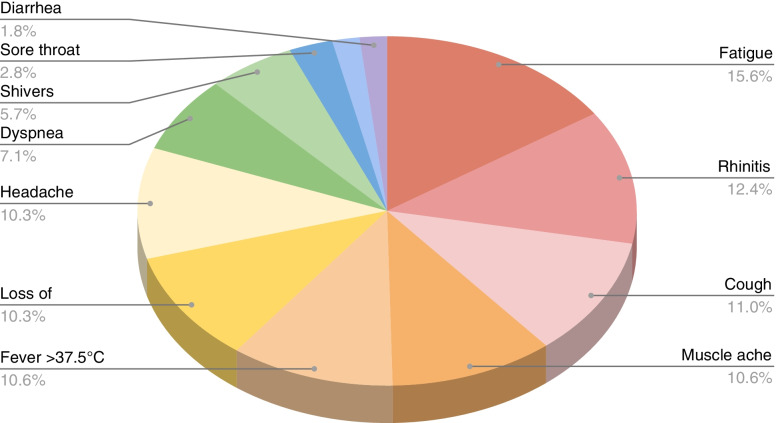
The most commonly reported symptoms of COVID-19 among infected women

The study involved 72 women during the 3rd wave of pandemic (15 February- 1 May 2021) of which 54 had recovered from a novel coronavirus infection at various trimesters of their pregnancy, 18 women with active infection during delivery and 17 healthy controls. Among 72 mothers with confirmed SARS-CoV-2 infection only 12.5% were asymptomatic. Sixty-three women (87.5%) had symptomatic COVID-19 infection; 59 of women manifested mild to moderate symptoms and 4 had severe infection symptoms. The most commonly reported symptoms were: fatigue (15.6%), rhinitis (12.4%), cough (11.0%), muscle ache (10.6%), fever (10.6%), loss of smell/taste and headache (10.3%) (Fig. 1).

The median age for all women analyzed in the study was 32 years. Seventeen pregnant women admitted to the hospital due to COVID-19 infection were over 35 years of age, which constituted 23.6% of the women participating in the study. Pre-existing diseases were found in 27 (37.5%) women patients. The most commonly reported chronic disorders were: thyroid diseases – 61.5%, insulin resistance – 23.1%, hypertension – 11.5%, endometriosis, PCOS – 7.7%, thrombophilia, thrombocytopenia, asthma, psoriasis, multiple sclerosis, connective tissue disorder – 3.8%. Pregnancy related pathologies were marked in 33.3% of the patients, out of which thyroid disorders – 62.5%, gestational diabetes – 20.8%, anemia – 20.8%, thrombocytopenia – 8.3% were mostly reported. Women participating in the study were mainly in their first pregnancy (47.2%). The mode of delivery was mostly caesarean section (CS) – 65.3%. Median gestational age at the delivery was 39 weeks. 76.4% deliveries occurred at term and 23.6% were preterm births. One mother (1.4%) was admitted to the Intensive Care Unit and required mechanical ventilation because of respiratory insufficiency associated with hypoxemic bilateral COVID-19 pneumonia. All women were discharged home recovered, with no acute symptoms of infection. No maternal nor neonatal deaths due to COVID-19 infection were reported. Two positive SARS-CoV-2 test results were registereded among infants born to COVID-19 mothers (2.3%). Neonates were born in good condition, median Apgar score was 10 at 1 and 5 minutes of life in full-term neonates and 8 and 9 at 1 and 5 minutes of life in preterm neonates respectively. Among 72 newborns of the study group, 17 were born prematurely (23.6%), no ones required resuscitation but 12 (16.6%) neonates needed „supporting transition” in the delivery room and 8 (11.1%) of them were admitted to the NICU with transient tachypnoe of newborn or respiratory distress syndrome due to birth before term, not symptoms of COVID-19 infection. Out of 72 newborns, 41 (56.9%) were exclusively breastfed and 31 (43.1%) were additionally fed with formula or donor milk from Human Milk Bank. Additional characteristics of study participants and their infants are presented in Table 1.

**Table 1:** Characteristics of study participants and their infants

Feature	Study Group ***n*** = 72	Control Group ***n*** = 17	***p*** value
**Age (years)**	32.5 ± 3.8	34.4 ± 4.2	0.082
	(32, 30-35)	(33, 32-37)	
**Gravida**			0.701
**1**	34 (47.2%)	7 (41.2%)	
**2**	30 (41.7%)	6 (35.3%)	
**≥ 3**	8 (11.1%)	3 (17.6%)	
**Body Mass Index (kg/m2)**	25.22 ± 3.83 (24.7, 22.8-27.6)	24.89 ± 3.86 (24.2, 22.1-28.4)	0.734
**Pre-existing comorbidities**	27 (37.5%)	5 (29.4%)	0.532
**Pregnancy morbidities**	38 (52.8%)	9 (52.9%)	0.535
**Mode of Delivery**			0.344
**vaginal**	25 (34.7%)	8 (47.1%)	
**caesarean section**	47 (65.3%)	9 (52.9%)	
**History of miscarriage prior to the current pregnancy**	22 (30.6%)	5 (29.4%)	.927
**Gestational age at birth (weeks)**	37.9 ± 2.9	37.6 ± 4.0	0.804
	(39, 37-40)	(39, 38-40)	
**Preterm birth**	17 (23.6%)	3 (17.6%)	0.596
**Child gender (F/M)**	33 (45.8%) / 39 (54.2%)	7 (41.2%) / 10 (58.8%)	0.7292
**Child weight (g)**	3233.7 ± 675.6	3291.9 ± 1174.8	0.208
	(3360, 2950-3700)	(3530, 3100-4140)	
**Child length (cm)**	51.3 ± 5.7	53.8 ± 7.6	**0**.**007**
	(52, 51-54)	(55, 53-58)	
**Apgar score**			
**≥ 37 weeks of GA**			
**1’**	10, 9-10	10, 10-10	**0.117**
**5’**	10, 10-10	10, 10-10	**0.298**
**< 37 weeks of GA**			
**1’**	8, 7-9	Data missed	
**5’**	9, 8-10	Data missed	
**Breastfeeding status**			0.108
**Only breast**	41 (56.9%)	6 (35.3%)	
**Mixed**	31 (43.1%)	11 (64.7%)	
**Symptoms of COVID-19**	62 (86.1%)	0 (100%)	**< 0**.**001**

Data are presented as n (%) or mean ± SD (median, IQR)

### IgA and IgG serum antibodies

**Fig. 2:**
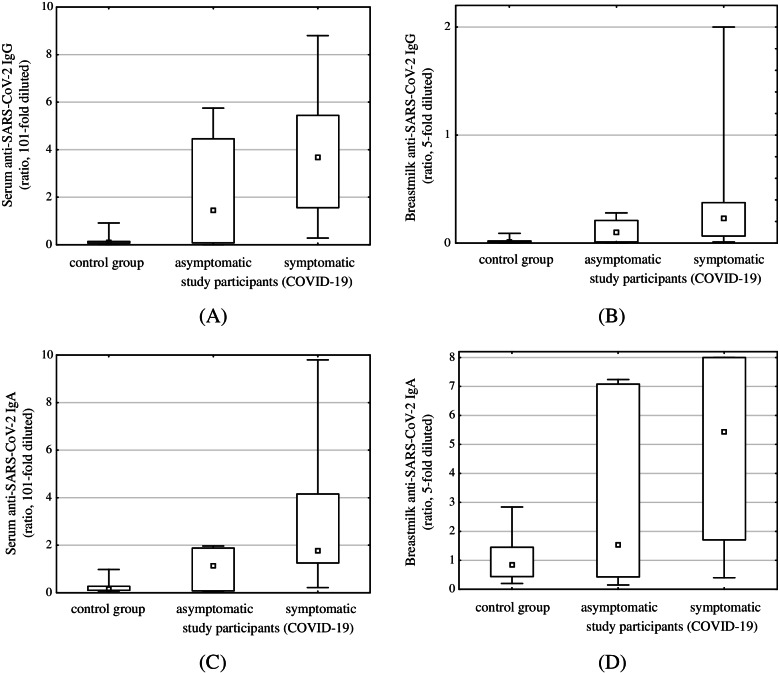
Serum and breastmilk anti-SARS-CoV-2 antibodies levels in relation to former SARS-CoV-2 exposure (asymptomatic or symptomatic). Levels of anti-SARS-CoV-2 IgG and IgA antibodies in serum and breastmilk were highly associated with former virus exposure (*p* <  0.001). No statistically significant difference in any antibody level was observed between asymptomatic and symptomatic COVID-19 study participants

Serum samples were available for 37 study participants and all controls. Anti-SARS-CoV-2 IgG antibodies were detected in 30 (81.1%) of COVID-19 convalescents (Table 2). IgA antibodies were detected in the sera of 28 (75.7%) mothers with former COVID-19. The prevalence and level of anti-SARS-CoV-2 IgG and IgA antibodies in serum were highly associated with former virus exposure (*p* <  0.001, Fig. 2).

**Table 2:** Anti-SARS-CoV-2 IgG and IgA antibody levels in the mothers’ serum samples

Antibody	Study Group	Control Group	***p*** value
IgG [ratio]	3.613 ± 2.480	0.143 ± 0.190	**< 0.001**
	(3.39, 1.45-5.38)	(0.10, 0.07-0.13)	
positive samples %	30 (81.1%)	0 (0%)	
IgA [ratio]	2.816 ± 2.836	0.222 ± 0.226	**< 0.001**
	(1.74, 1.11-3.05)	(0.15, 0.10-0.27)	
positive samples %	28 (75.7%)	0 (0%)	

Data are presented as mean ± SD (median, IQR). Serum samples were assayed 101-fold diluted

**Fig. 3:**
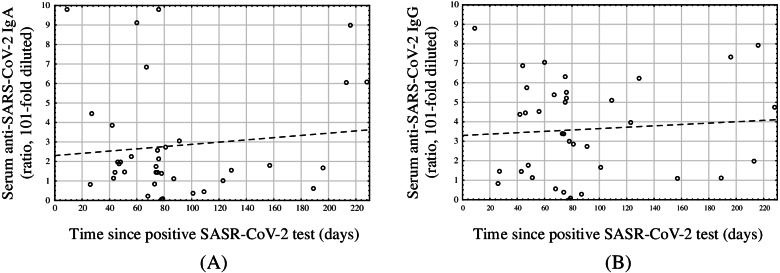
Correlation between serum SARS-CoV-2 IgG and IgA levels and time since positive SARS-CoV-2 test result. No statistically significant correlation was observed (IgG: rs = −0.01, p = 0.936; IgA: rs = − 0.10, p = 0.544; time up to 229 days)

We did not observe a statistically significant difference between the antibodies levels in serum samples obtained from symptomatic and asymptomatic SARS-CoV-2 exposed women (IgG: *p* = 0.197; IgA: *p* = 0.981). Moreover, there was no statistically significant correlation between antibodies levels and time since positive SARS-CoV-2 test in the timespan studied (IgG: rs = − 0.01, *p* = 0.936; IgA: rs = − 0.10, *p* = 0.544; time up to 229 days) (Fig. 3). No relation of the antibodies levels and the trimester of the pregnancy at COVID-19 was observed (Supplementary Table S1).

### IgA and IgG human milk antibodies

**Fig. 4:**
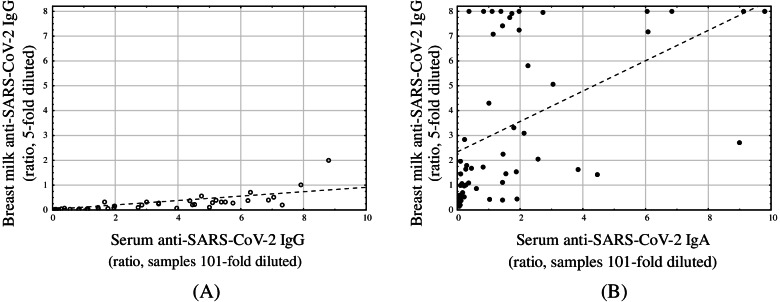
Correlation between breastmilk and serum antibody levels (IgG: rs = 0.92, *p* <  0.001; IgA rs = 0.67, *p* <  0.001)

Table 3 summarizes the levels of anti-SARS-CoV-2 IgG and IgA antibodies detected in breastmilk samples obtained from the study participants (IgG: 0.25, 0.00–0.55 BAU/ml, ratio 0.02, 0.01–0.04; IgA: ratio 7.52, 2.68–8.00) and controls (IgG: all < 0.16 BAU/ml, ratio 0.01, 0.01–0.02; IgA: ratio 0.99, 0.53–1.45). The breastmilk samples obtained from women with formerly confirmed COVID-19 presented higher concentrations of antibodies compared to controls (*p* <  0.001). Moreover, the breastmilk samples obtained from seropositive study participants presented higher (*p* <  0.001) concentration of anti-SARS-CoV-2 IgG (0.53, 0.18–0.66 BAU/ml, ratio 0.14, 0.06–0.32) and IgA (ratio 7.12, 2.15–8.00) compared to seronegative participants (IgG: all < 0.16 BAU/ml, ratio 0.02, 0.01–0.04; IgA: ratio 1.68, 0.86–4.30). The levels of antibodies in serum and breastmilk samples were highly correlated (IgG: rs = 0.92, *p* <  0.001; IgA rs = 0.67, *p* <  0.001, Fig. 4).

**Table 3:** Anti-SARS-CoV-2 IgG and IgA antibody levels in breastmilk

Antibody	Study Group	Control Group	***p*** value
IgG [BAU/ml]	0.366 ± 0.544	0.00	**< 0.001**
	(0.25, 0.00-0.55)		
number of samples below detection limit of an assay	28 (38.9%)	17 (100%)	
IgG [ratio]	0.226 ± 0.283	0.014 ± 0.011	**< 0.001**
	(0.14, 0.06-0.32)	(0.01, 0.01-0.02)	
IgA [ratio]	5.588 ± 2.906	1.029 ± 0.710	**< 0.001**
	(7.52, 2.68-8.00)	(0.99, 0.53-1.45)	
number of samples above IgA assay range	31 (43.1%)	0 (0.0%)	

Data are presented as mean ± SD (median, IQR). IgG level for samples below detection limit were assigned 0 BAU/ml. IgA level for samples exceeding assay range was assigned a ratio of 8. Breast milk was assayed 5-fold diluted

The breastmilk samples of the study participants were characterized by a higher level of anti-SARS-CoV-2 IgA antibodies (ratio 5.06, 1.63–8.00) compared to IgG (ratio 0.21, 0.06–0.32; *p* < 0.001).

ROC analysis revealed that detection of breastmilk IgG >  0.16 BAU/ml (ratio >  0.04) and IgA > with ratio >  1.96 are strongly related to former SARS-CoV-2 exposure (Table 4).

**Table 4:** ROC analysis results – breastmilk antibody levels in relation to former COVID-19

Antibody	AUC	***p*** value	Cutoff	Sensitivity	Specificity
IgG [BAU/ml]	0.806	< 0.001	> 0.16	59.7%	100%
IgG [ratio]	0.931	< 0.001	> 0.04	81.9%	100%
IgA [ratio]	0.909	< 0.001	> 1.96	79.2%	94.1%

We did not observe any statistically significant difference between the antibodies levels in breastmilk samples obtained from symptomatic and asymptomatic SARS-CoV-2 exposed women (IgG: *p* = 0.547; IgA: *p* = 0.097). Moreover, there was no statistically significant correlation between antibodies levels and time since positive SARS-CoV-2 test over the timespan studied (IgG: rs = 0.15, *p* = 0.223; IgA: rs = 0.06, *p* = 0.599; time up to 229 days). No relation of the antibodies levels and the trimester of the pregnancy at COVID-19 was observed (Supplementary Table S1).

## Discussion

The paper describes a group of 72 mothers recovered from COVID-19 during the 3rd wave of pandemic. The study included women with a different time window between the COVID-19 infection and time of sampling. Therefore investigation over the antibody levels dynamics over time was possible. Human milk is a source of antigen-specific antibodies. IgA is the predominant antibody isotype in human milk (approximately 90%) nearly all in secretory (s) form (sIgA). These antibodies are responsible for conferring mucosal immunity and passive transfer during breastfeeding protecting infants from potential pathogens [8, 9].

We have detected the presence of IgA antibodies against the virus in 62 out 72 patients (86.1%) in the breast milk samples. Low et al. in a systematic review from 2021 gathered the data of 161 lactating individuals with COVID-19 infection. According to this study human milk from 69 of 86 individuals (80.2%) contained SARS-CoV-2-specific IgA [10]. Specific IgA to SARS-CoV-2 was found in 80% of samples in the study of Fox et al. (mostly being secretory IgA) [5] in 97% of samples in the study of Demers-Mathieu et al .[11] and in 76% of samples in Pace et al. study [6]. In our study all sera samples (*n* = 18) collected up to 7 days postpartum already contained specific IgA antibodies. Lebrão et al. demonstrate positive IgA at 3 and 6 days after delivery in one mother [12]. In our study we also found level of antibodies in one individual persisting up to 8 months after the infection (229 days). However, median durability of human milk antibody production could not be reported due to heterogeneity of reporting. Based on systematic review by Low et al. [10] the longest duration of antibody persistence in human milk reported from onset of COVID-19 infection until end of study was 195 days in a single individual [13]. Duncombe et al. reported durable anti-RBD IgA antibodies in breast milk persisting over 6 months [14] and recent study from Juncker et al. reported that IgA antibodies in human milk remain present at least 10 months after PCR confirmed infection [15].

In our study anti-SARS-CoV-2 IgG antibodies were detected in 61 out of 72 (84.7%) breastmilk samples. The breastmilk samples obtained from women with formerly confirmed COVID-19 presented higher concentrations of SARS-CoV-2 IgG and IgA compared to controls (*p* < 0.001). Moreover, the breastmilk samples obtained from seropositive study participants presented higher (*p* < 0.001) concentration of anti-SARS-CoV-2 IgG and IgA compared to seronegative. In our study the concentrations of anti-SARS-CoV-2 IgA antibodies were consistently higher than those of IgG antibodies. Our findings are consistent with Pace et al. study where all samples of recently diagnosed women comprised IgA and IgG antibodies against the virus and their levels were higher than in milk collected from women before pandemic. Observed anti-RBD-IgA levels were also higher than IgG antibodies [6]. In fact, it has been demonstrated that for SARS-CoV- 2, the majority of antibodies (60%) are IgA or secretory IgA, which may explain higher IgA levels [16]. Moreover, only a small fraction of milk antibodies originates from serum- IgG comprise only 2% of milk Ab [8].

Besides milk samples we managed to collect additionally 55 serum samples. Anti-SARS-CoV-2 IgG antibodies were detected in the sera of 81.1% mothers with former COVID-19 and anti-SARS-CoV-2 IgA antibodies in 75.7% samples. The serum of women with COVID-19 was characterized by a higher concentration of anti-RBD IgA and IgG than the serum from the control group without COVID-19 with statistical significance of *p* < 0.001 for both antibodies. A statistically significant positive correlation was found between the values of IgG in milk and maternal serum (rs = 0.92, *p* < 0.001) and of IgA in milk and maternal serum (rs = 0.67, *p < 0.001*). Levels of SARS-CoV-2 IgA were higher in human milk than in serum samples, regarding SARS-CoV-2 IgG- their levels in serum were higher than in human milk. Our findings are in accordance with Dong et al. study, which showed that the serum SARS-CoV-2- specific IgG level was higher (292 to 2093 fold) than human milk SARS-CoV-2-specific IgG from one mother with confirmed SARS-CoV-2 infection [17]. Demers-Mathieu et al. demonstrated differences between human milk and serum samples from COVID-19-recovered women. When the AUCs were not divided by the antibody concentration, SARS-CoV-2 RBD-specific IgA and IgG levels were higher in serum than the human milk. However, diving AUC by antibody concentrations demonstrated SARS-CoV-2 RBD-specific IgG (AUC/lgG) higher in human milk than in serum (*p* < 0.05), whereas, SARS-CoV-2 RBD-specific IgA (AUC/mg of IgA) was higher in the serum than the human milk (*p* < 0.01) [16].

Neutralization capacity tends to correlate with RBD-specific antibody levels in various serological studies [18]. Although our study did not evaluate the neutralization capacity of IgG and IgA, previous studies showed that human milk can neutralize SARS-CoV-2 in vitro [6, 13, 19]. Samples from 20 of 48 individuals (41.7%) – data from systematic review were found to neutralize SARS-CoV-2 infectivity in vitro [10]. Further investigations are warranted to evaluate protective capacity of human milk and whether lactating mothers who have recovered from COVID-19 transfer protective antibodies to their nursing infants. Regarding human milk banking neutralizing capacity of human milk was also evaluated after Holder pasteurization and high-pressure pasteurization. After Holder pasteurization SARS-CoV-2 IgA levels remained unchanged and the neutralizing function was significantly reduced or lost. Regarding high-pressure pasteurized-treated milk antibodies remain effective at neutralizing the virus [20].

## Conclusion

In conclusion, our study provided evidence that SARS-CoV- 2 IgA and IgG antibodies are present in the milk of women who have recovered from COVID-19 and last up to 8 months post-infection. Their presence may be related with viral immunity, which can be passed on to breastfeeding infants and serve as a protection against COVID-19 and/or severity of the disease. Understanding the dynamics of antibodies in breast milk is urgently needed to improve our knowledge whether a typical response is truly protective for breastfed babies.

## Supplementary Information

**Additional file 1: Table S1.** Anti-SARS-CoV-2 IgG and IgA antibody levels in the mothers’ serum samples and breastmilk samples in relation to the time of SARS-CoV-2 infection.

## Data Availability

The datasets analysed during the current study are available in supplementary information files. Data regarding participants of the study are available from the corresponding author on reasonable request after de- identify.
