# Supplementary material for: Antibodies in the breastmilk of COVID-19 recovered women
Source: BMC Pregnancy Childbirth. 2022 Aug 11;22:635. doi: 10.1186/s12884-022-04945-z (PMC9368700; doi:10.1186/s12884-022-04945-z)
Supplement: Supplementary file 1 — Additional file 1: Table S1. Anti-SARS-CoV-2 IgG and IgA antibody levels in the mothers’ serum samples and breastmilk samples in relation to the time of SARS-CoV-2 infection. [file 12884_2022_4945_MOESM1_ESM.docx]

Supplementary Table 1

**Table S1.** Anti-SARS-CoV-2 IgG and IgA antibody levels in the mothers’ serum samples and breastmilk samples in relation to the time of SARS-CoV-2 infection

| **Antibody** | **1st trimester** | **2nd trimester** | **3^rd^ trimester (incl. during delivery)** | **p value** |
| --- | --- | --- | --- | --- |
| Serum IgG [ratio] | 3.998 2.361  3.35, 2.36 5.64 | 2.945 1.926  2.96, 1.29 4.61 | 3.652 2.612  3.39, 1.13 5.51 | 0.820 |
| Serum IgA [ratio] | 2.945 2.238  2.36, 1.34 4.55 | 3.280 2.455  3.22, 1.26 5.27 | 2.734 3.019  1.55, 1.11 2.57 | 0.657 |
| Breastmilk IgG [BAU/ml] | 0.267 0.227  0.27, 0.00 0.41 | 0.346 0.356  0.28, 0.00 0.66 | 0.389 0.612  0.20, 0.00 0.55 | 0.938 |
| Breastmilk IgG [ratio] | 0.169 0.095  0.15, 0.09 0.20 | 0.220 0.182  0.16,0.08 0.34 | 0.238 0.320  0.12, 0.05 0.32 | 0.890 |
| Breastmilk IgA [ratio] | 6.63 1.88  8.00, 4.30 8.00 | 6.078 3.070  7.59, 4.30 8.00 | 5.298 3.040  7.34, 2.05 8.00 | 0.390 |

Data are presented as mean ±SD (median, IQR). IgG level for samples below detection limit were assigned 0 BAU/ml. IgA level for samples exceeding assay range was assigned a ratio of 8. Breastmilk was assayed 5-fold diluted. Serum samples were assayed 101-fold diluted.
